# Sickness absenteeism among health workers in Cuiabá, Brazil

**DOI:** 10.47626/1679-4435-2022-744

**Published:** 2023-02-03

**Authors:** Rita Adriana Gomes de Souza, Lucia Helena Frasson

**Affiliations:** 1 Collective Health Institute, Universidade Federal de Mato Grosso, Cuiabá, MT, Brazil

**Keywords:** absenteeism, occupational diseases, occupational health, health personnel, absenteísmo, doenças profissionais, saúde do trabalhador, pessoal de saúde

## Abstract

**Introduction:**

Sickness absenteeism has been considered a complex phenomenon, with multiple etiologies, including factors related to both the environment and the organization of work, as well as individual factors. However, it has been studied in restricted occupational groups.

**Objectives:**

To analyze the profile of sickness absenteeism among workers of a health company in Cuiabá, state of Mato Grosso, Brazil, in 2015 and 2016.

**Methods:**

Cross-sectional study, with workers present in the company’s payroll from 01/01/2015 to 12/31/2016, with a medical certificate approved by the occupational physician to justify absence from work. The variables analyzed were the chapter of the disease according to the International Statistical Classification of Diseases and Health Problems, sex, age, age group, number of medical certificates, days of absenteeism, the section of work activities, function performed at the time of sick leave, and indicators related to absenteeism.

**Results:**

3,813 sickness leave certificates were registered, which corresponded to 45.4% of the company’s workers. The mean number of sickness leave certificates was 4.0, which led to 18.9 days of absenteeism on average. The highest percentages of sickness absenteeism were found in women, in those with diseases of the musculoskeletal system and connective tissue, in those working in Emergency Room sections and in the roles of customer service agents and analysts. Considering the longest periods of absence, the most identified categories were older people, circulatory system diseases, work in the administration section, and the motorcycle courier position.

**Conclusions:**

A considerable percentage of sickness absenteeism was identified in the company, requiring managers to invest in strategies to adapt the work environment.

## Abstract (pt)

**Introdução:**

O absenteísmo-doença tem sido considerado um fenômeno complexo e de etiologia múltipla, incluindo fatores relacionados tanto ao ambiente e à organização do trabalho quanto a fatores individuais. No entanto, ele tem sido estudado em grupos ocupacionais restritos.

**Objetivos:**

Analisar o perfil de absenteísmo-doença em trabalhadores de uma prestadora de serviços na área da saúde em Cuiabá, no estado de Mato Grosso, em 2015 e 2016.

**Métodos:**

Estudo transversal, com trabalhadores presentes na folha de pagamento da empresa de 01/01/2015 a 31/12/2016, com atestado médico homologado pelo médico do trabalho para justificar a ausência ao trabalho. Foram analisadas as seguintes variáveis: capítulo da doença, sexo, idade, faixa etária, número de atestados, dias de afastamento, seção de exercício das atividades laborais, função exercida no momento do afastamento e indicadores relacionados ao absenteísmo.

**Resultados:**

Foram registrados 3.813 atestados médicos, abrangendo 45,4% dos trabalhadores da empresa. A média de atestados foi de 4,0, que gerou, em média, 18,9 dias de afastamento. Os maiores percentuais de absenteísmo-doença foram encontrados nas mulheres, naqueles com doenças do sistema osteomuscular e do tecido conjuntivo, nas seções do Pronto Atendimento e nas funções de agentes de atendimento e analistas. Considerando os maiores tempos de afastamento, esses foram identificados nos mais velhos, naqueles com doenças do aparelho circulatório, na seção de administração e na função de motoboy.

**Conclusões:**

Identificou-se percentual considerável de absenteísmo-doença na empresa, sendo necessário que os gestores invistam em estratégias para adequação do ambiente de trabalho.

## INTRODUCTION

Despite being a social organizer that invests social actors with identity, work is also responsible for contributing significantly to the workers’ illness. Therefore, absence from work could be a form of resistance to the demands of an unhealthy job, representing a form o resistance and escape from the situation in which one finds oneself.^1^

Absenteeism, characterized as employee absence from work, has been considered a complex phenomenon with multiple etiologies, including factors related to the work environment and organization, as well as individual issues. When the absence is due to an illness or grievance certified by medical leave, it is usually called sickness absenteeism, and it is considered an essential indicator of the institutions’ working conditions and workers’ health.^2^

Once it requires the resizing of the workforce, increasing production or service delivery costs, and consequently increasing the final cost of the product or service, absenteeism has a significant economic impact. Institutions’ concerns are often motivated by financial expenses and decreased productivity; however, the phenomenon cannot be seen solely as a socioeconomic concern.^3^

In Brazil, the levels of sickness absenteeism vary significantly according to the institution, the administration, and the type of activity performed, and epidemiological studies on this phenomenon have been mainly restricted to specific occupational groups, such as nurses and teachers.^4,5^ Therefore, there is a need to study the epidemiological profile and the occurrence of such absences in other work environments and categories.

The analysis of events related to the work environment, specifically those associated with absence from work for health-related reasons, allows situational diagnoses and interventions to promote and prevent worker health and is, thus, relevant for workers’ health surveillance and from an administrative perspective.^6^

Therefore, this research aimed to analyze the profile of sickness absenteeism of workers at a healthcare service provider in the municipality of Cuiabá, state of Mato Grosso, in 2015 and 2016.

## METHODS

This was a cross-sectional study, conducted with workers at a healthcare service provider located in the municipality of Cuiabá, state of Mato Grosso, in the years 2015 and 2016.

In the study period, the healthcare service provider comprised 133 clinics, 21 hospitals, 51 accredited laboratories (37 accredited as legal entities), and 7 hospitals in the state’s countryside, assisting around 220 thousand people. At a local level, it was considered the largest company in the industry and, at a national level, it figures as the largest health care network in Brazil, present in 83% of the national territory.

In the service provider’s collective agreement, there is a clause establishing a 48-hour deadline for delivering sick leave certificates to the Human resources management sector, which must be signed by the immediate leader and the worker. The certificate is then directed to the occupational safety sector, responsible for entering the data into the company’s internal control system, making it possible to issue reports. In the present study, we analyzed the data from the certificates, which feed the company’s occupational health service database.

Participants in this study included workers hired by direct employment via a selection process, who were on the payroll from 01/01/2015 to 12/31/2016 and who presented a medical sickness leave certificate during this period to justify absence from work. The medical leave certificates issued by occupational physicians were considered.

The family medical leaves issued to care for a family member and childbirth were excluded, since they are not configured as causes for treatment of the worker’s health, despite originating from medical leave certificates.^7^

The study outcome, sickness absenteeism, was considered the absence from work related to illness justified by medical certificate.

The following variables related to sickness absenteeism were considered: disease chapter (reason for the medical certificate) according to the 10th revision of the International Statistical Classification of Diseases and Health Problems (ICD-10), sex (women and men), age, age group according to tertiles (15 to 29 years, 30 to 36 years, and 37 to 73 years), number of certificates, days away from work, the section where the employee conducted his/her work activities (emergency room; pharmacies; customer services, distribution of medicines, and home care services; financial; administration; variable costs; occupational health and physiotherapy center; clinical care; information technology center; exchange center; others) and the role held by the employee at the time of leave (customer service agent; analyst; telemarketing; assistant; technician; assistant; stocker; courier; nurse; supervisor; others).

In addition to these variables, the following indicators proposed by the Permanent Commission and International Association on Occupational Health^8^ were calculated a) frequency of sick leave: number of certificates in the period/average effective in the period; b) frequency of workers on sick leave: number of workers on sick leave in the period/average effective in the period; c) severity rate: number of days lost in the period/average effective in the period; and d) duration of absenteeism: total number of days of sick leave/number of episodes of sick leave, proposed by Hensing et al.^9^

Descriptive measures were provided according to the variable type, such as absolute and relative frequencies, mean, and standard deviation. The Mann-Whitney test was used to test the difference between the means. Significance was established at 5% (p < 0.05). Statistical analyses were performed using the IBM SPSS, version 22.

This study was approved by the Research Ethics Committee of the Universidade Federal de Mato Grosso under number 2.420.070 and complied with the research ethics recommendations of the Declaration of Helsinki and the standards established in Resolutions No. 466/2012 and No. 510/2016 of the Brazilian National Health Council.

## RESULTS

During the study period, a total of 3,813 medical certificates were recorded in the healthcare provider’s occupational health system. With an average of 1,065 employees/year, the mean frequency rate of sick leave was 1.79. In 2015, these certificates represented 10,626 days of absence; in 2016, they represented 7,493 days, totaling a mean severity index of 8.51 and absenteeism duration of 4.75. The certificates that presented up to 3 days of absence represented 39.6% of the total. In 2015, 373 women (74.5%) and 128 men (25.5%) had presented at least one medical certificate; in 2016, there were 324 women (69.8%) and 140 men (30.2%). These contingents represent about 45.4% of employees in the company, i.e., a frequency rate of workers on sick leave of 0.45. The overall mean age was 33.7 years (SD, 8.7), with no statistically significant difference between the sexes (data not shown in tables).

The mean number of medical certificates presented per worker on sick leave was 4.0, and these certificates led to a mean of 18.9 days of absence. Women presented 1.5 times more certificates than men (p < 0.05), and older people (37 to 73 years old) presented more days of absence than the other age groups (p < 0.05). There was no statistically significant difference in the number of certificates according to age group and days away from work according to sex (Table 1).

**Table 1:** Number of medical certificates and days off related to sickness absenteeism according to sex and age group in workers of a health care service provider, Cuiabá, 2015 and 2016

Number of medical certificates and days off	Mean	Standard deviation
Number of medical certificates and days off^*^		
General	4.0	3.6
Women	4.4	3.9
Men	2.9	2.4
Number of medical certificates (age range)		
15 to 29	4.1	3.8
30 to 36	3.8	3.1
37 to 73	4.0	3.9
Days off work (sex)		
General	18.9	44.3
Women	19.1	41.0
Men	18.4	51.9
Days off work (age group)^*^		
15 to 29	15.4	37.7
30 to 36	19.3	48.4
37 to 73	22.4	46.3

* p < 0.05.

Of the total number of medical certificates submitted in 2015 and 2016 (n = 3,813), only 1,305 (34.2%) included the ICD-10 chapter. The diseases most commonly recorded in medical certificates are shown in Table 2. Diseases of the musculoskeletal system and connective tissue represented the first cause of sickness absenteeism (20.9%). These diseases, along with symptoms, signs, and abnormal findings of clinical and laboratory tests (not classified elsewhere), respiratory system diseases, injuries, poisonings, and some other consequences of external causes, accounted for 55.5% of the diseases recorded in medical certificates.

**Table 2:** Diseases according to the chapters of the 10th revision of the International Statistical Classification of Diseases and Related Health Problems (ICD-10) and days of sick leave among workers of a healthcare service provider, Cuiabá, 2015 and 2016

Diseases according to ICD-10 codes	n	%	Days off work (mean)
Diseases of the musculoskeletal system and connective tissue	273	20.9	7.5
Symptoms, signs, and abnormal findings of clinical and laboratory examinations not elsewhere classified	207	15.9	4.2
Diseases of the respiratory system	138	10.6	4.2
Injuries, poisoning, and other consequences of external causes	106	8.1	23.6
Diseases of the ear and mastoid apophysis	85	6.5	3.3
Certain infectious and parasitic diseases	76	5.8	3.4
Diseases of the genitourinary system	72	5.5	5.3
Diseases of the circulatory system	59	4.5	29.3
Diseases of the digestive system	55	4.2	7.8
Complications related to pregnancy, childbirth, and puerperium	48	3.7	20.6
Other	186	14.3	5.3

In the group of diseases of the osteomuscular and connective tissue systems, joint pain was the most frequent (26.7%), followed by low back pain (13.6%), and synovitis and tenosynovitis (9.2%). In the group of symptoms, signs, and abnormal findings of clinical and laboratory examinations (not elsewhere classified), abdominal and pelvic pain (52.2%) and headache (8.2%) were the most frequently recorded. In the group of respiratory system diseases, sinusitis was the most frequent (34.1%), followed by tonsillitis (21%). In the group of injuries, poisoning, and some other consequences of external causes, 20.8% of the records presented knee-related diseases (data not shown in tables).

The diseases recorded as circulatory system diseases were those that presented, on average, the longest time of absence from work (29.3 days), followed by diseases in the group of injuries, poisoning, other consequences of external causes (23.6 days), and complications related to pregnancy, childbirth and puerperium (20.6 days) (Table 2).

The distribution of diseases according to sex is presented in Table 3. There is a difference in the order of importance of disease categories between men and women, and women’s certificates showed a higher proportion of ICD-10 registered. In women, diseases of the musculoskeletal system and connective tissue, symptoms, signs and abnormal findings of clinical and laboratory tests (not classified elsewhere), and diseases of the respiratory system were the most frequently recorded, representing 47.9% of the total. For men, diseases of the musculoskeletal system and connective tissue, injuries, poisoning, and other consequences of external causes and diseases of the respiratory system accounted for 54.6% of the diseases recorded in medical certificates.

**Table 3:** Diseases according to the chapters of the 10th revision of the International Statistical Classification of Diseases and Related Health Problems (ICD-10) related to sickness absenteeism, according to sex, among workers at a health service provider in Cuiabá, 2015 and 2016

Diseases according to the ICD-10 chapters	n	%
Women		
Diseases of the musculoskeletal system and connective tissue	211	20.0
Symptoms, signs and abnormal findings on clinical and laboratory examinations, not elsewhere classified	185	17.6
Diseases of the respiratory system	109	10.3
Diseases of the eye and its appendages	70	6.6
Diseases of the genitourinary system	67	6.4
Injuries, poisonings, and other consequences of external causes	65	6.2
Some infectious and parasitic diseases	52	4.9
Complications related to pregnancy, childbirth, and puerperium	48	4.6
Diseases of the circulatory system	47	4.5
Diseases of the digestive system	40	3.8
Other	160	15.2
Total	1.054	100.0
Men		
Diseases of the musculoskeletal system and connective tissue	62	24.7
Injuries, poisoning, and some other consequences of external causes	46	18.3
Respiratory system diseases	29	11.6
Certain infectious and parasitic diseases	29	11.6
Symptoms, signs, and abnormal findings on clinical and laboratory examinations, not elsewhere classified	22	8.8
Diseases of the ear and mastoid apophysis	15	6.0
Diseases of the digestive system	15	6.0
Diseases of the circulatory system	12	4.8
Diseases of the genitourinary system	5	2.0
Other	16	6.4
Total	251	100.0
Overall Total	1.305	100.0

It is noteworthy that the diseases of the eye and its appendages ranked fourth as the most recorded category of diseases in women, and complications related to pregnancy, childbirth, and the puerperium appeared in the eighth position. In men, diseases of the ear and mastoid apophysis ranked sixth, appearing only in this group (Table 3).

Table 4 shows the sections whose workers presented the highest number of medical certificates. The emergency service, pharmacy, and customer-relations sections were responsible for 51.8% of the sick leaves. On the other hand, the administration (32.2 hours), emergency service (24.3 hours), pharmacies (24.3 hours), and occupational health and physical therapy sections (24.3 hours) were the ones whose workers had the longest sick leaves.

**Table 4:** Sickness absenteeism according to work section and job performed in workers of a health care service provider, Cuiabá, 2015 and 2016

Work section	n	%	Days of sick leave (mean)
Emergency service	182	18.9	24.3
Pharmacies	161	16.8	24.3
Customer service	155	16.1	16.4
Medication distribution and home care services	87	9.1	19.8
Financial	75	7.8	10.9
Administration	60	6.2	32.2
Variable costs	47	4.9	12.0
Occupational health and physical therapy care	45	4.7	24.3
Care Clinic	32	3.3	10.7
Information Technology Center	22	2.3	5.3
Exchange Center	22	2.3	10.0
Other	73	7.6	7.4
Total	961	100.0	
Role performed			
Service agent	133	13.9	19.0
Analyst	129	13.4	11.7
Telemarketing	119	12.4	14.7
Auxiliary	116	12.1	32.8
Technician	69	7.2	12.9
Assistant	68	7.1	15.0
Stocker	53	5.5	21.9
Motorcycle courier	48	5.0	45.3
Nurse	38	4.0	12.1
Supervisor	24	2.5	15.1
Other	163	17.0	20.0
Total	960	100.0	

Service agents, analysts, telemarketing workers, and assistants accounted for 51.8% of the jobs that presented more medical certificates, while motorcycle couriers, assistants, and stockers presented the longest absenteeism/sick leave, 45.3 days, 32.8 days, and 21.9 hours, respectively (Table 4).

## DISCUSSION

This study presents the results of the analysis of sickness absenteeism in a healthcare service provider located in Cuiabá, state of Mato Grosso, in 2015 and 2016. This is the first study to analyze this event in a company of this size in the municipality.

The results showed a worrying percentage of sickness absenteeism in the company since about 45% of the workers presented a medical certificate as a justification for absence at least once. In total, 3,813 certificates were received during the study period, accounting for 18,119 days of absence, which is equivalent to saying that approximately 25 workers did not show up at the company during the study period. This quantity illustrates the loss that sickness absenteeism can represent for the institution.

Absenteeism is a highly complex problem because it involves intrinsic and extrinsic work factors,^10^ and studies have shown that absence for medical reasons is the most frequent among absences.^11,12^ Its consequences include work overload and dissatisfaction of the remaining workers, disorganization of teamwork, and a decrease in the quantity and quality of the work performed. Thus, the interests of the institution and the workers are affected, as well as the working relationship between them.^3^

In a cross-sectional study conducted with 3,403 workers from manufacturing, civil construction, and industrial public service companies located in the state of Bahia, the overall percentage of sickness absenteeism was 13.5% from 16 to 65 years.^13^ In the study by Barboza & Soler,^14^ which aimed at characterizing absences among nursing workers from a general teaching hospital in the municipality of São José do Rio Preto (state of São Paulo), it was found that health problems caused 75.4% of absences from work. In a one-year longitudinal study of 2,150 workers from a Brazilian airline company, 53.5% of the individuals had at least one episode of absence due to illness, and these sick leaves caused, on average, the loss of 8.3 working days.^15^ Therefore, the percentages of sickness absenteeism vary considerably among the populations studied.

In the present study, women presented the highest rates of sickness absenteeism, at least more than twice as much as men. In this aspect, we can consider that sex characteristics have shown an important role in triggering sickness absenteeism: women’s entry into the labor market, with low pay and a higher degree of exploitation, which causes higher rates of disease;^16^ the double working shifts (work and household chores), with a higher overload of activities on a daily basis;^17^ and the higher demand of women for health services, which, consequently, causes women to have more preventive health care than men, taking more time off work for this purpose.^18^

Although there was no difference in the number of certificates, the days away from work increased as age increased, i.e., older workers had longer time away from work due to absenteeism. Vivolo^19^ found a similar result in a study that evaluated absences due to health problems of workers at the Health Department of the state of São Paulo. According to Affonso, ^11^ older people have higher rates of non-preventable absences from work, perhaps due to more fragile health associated with aging and a longer recovery period when they need to be absent to take care of their health.

One must consider, additionally, other factors for absenteeism in older workers: reduced muscle strength, reduced hearing ability, altered psychomotricity with slower movements and reduced reach, altered memory with reduced recent memory, and reduced information retention capacity, which can cause difficulties in learning new technologies.^20^ Biological difficulties can generate conflict for the elderly worker, for having to deal with the efforts of adaptation and the need to remain in the labor market.

It was expected that not all medical certificates would have ICD-10 chapters, since this register is not mandatory, but the fact that only 34.2% of the medical certificates had this classification may have hindered the identification of diseases and illnesses that most affected the workers. In the study by Silva & Marziale,^21^ which investigated the sickness absenteeism in 199 nursing workers, 31% of the certificates did not include diagnosis or ICD-10. However, the profile of illness of certain groups of workers has indicated a high occurrence of diseases of the musculoskeletal system and connective tissue,^6,22^ which were also the most frequent diseases among the workers in this study.

Due to the changes that have occurred in the world of work, the worker is still being demanded, but in a different way. The efforts have been modified, becoming less intense from the strength perspective but more constant and faster. As a consequence of this new worker-work relationship, there is an environment more favorable to developing diseases related to repetitive work, to accelerated rhythms, and, consequently, to stress, such as diseases of the musculoskeletal system and connective tissue. This scenario can lead to a picture of illness characteristic of specific work processes and to the rates of sickness absenteeism observed in many institutions.^22^

Osteomuscular system and connective tissue diseases, along with symptoms, signs, and abnormal findings of clinical and laboratory tests (not classified elsewhere), respiratory system diseases, injuries, poisoning, and some other consequences of external causes, accounted for 55.5% of the groups of diseases recorded in medical certificates. A similar result was found in the study by Simões & Rocha,^23^ who evaluated 883 rural workers in 2009 in Minas Gerais and found that these diseases were also the most frequent in the study group, accounting for 54.8% of the causes recorded in the sickness leave certificates.

In general, it was found that the groups of diseases were the same in both sexes but differed in order of importance. Without considering complications related to pregnancy, labor, and puerperium, the differences were diseases of the eye and its appendages, which appeared only in women, and diseases of the ear and mastoid apophysis, which appeared only among men. In the study by Gomes,^24^ who evaluated absenteeism among nursing professionals in a university hospital in the municipality of Recife (state of Pernambuco), diseases of the eye and appendages and diseases of the ear and mastoid apophysis were among the 3 most frequent.

Eye, nose, and throat irritation complaints are frequent among workers as a consequence of exposure to harmful substances in the work environment, such as cleaning chemicals, to lighting, and to enclosed places with inadequate ventilation.^21^

Considering sickness absenteeism according to the section of the company, we verified that the sections most related to contact with the public presented the highest percentage. According to Serafim et al.^25^, the worker who is in direct and constant contact with the public functions as the “front line” of the institution and is, therefore, more susceptible to hearing users’ complaints. Moreover, the sections with direct contact with the public, along with the administration section, were those that presented the longest time away from work, which may suggest health conditions characterized as chronic.

Relating sickness absenteeism to the role in the health care service, we verified that it occurred more frequently among workers whose roles required less schooling. These workers also had more extended periods of sickness absenteeism. According to Costa et al.,^26^ elementary and high school level workers are absent from work more often than higher education level workers. In the mentioned study, which evaluated 565 sick leaves among nursing employees in a public hospital in Minas Gerais, 56.8% occurred among nursing assistants, 29.7% among nursing technicians, and only 13.5% among nurses. Other studies also corroborated the relationship between lower education levels and sickness absenteeism.^27,28^

Duties related to lower schooling also have lower pay, which can reflect in greater difficulty in adopting healthier habits, such as physical exercise and proper nutrition, in addition to less access to health services and good housing conditions, affecting health levels and, potentially, sickness absenteeism.^29^ Moreover, tasks that require less education may require more physical effort and repetitive activities and may be more monotonous.

According to Reis et al.^27^, the differences in schooling may also affect the registration of sickness absenteeism since absences are more easily solved by internal arrangements among hierarchically superior workers, which can lead to a lower registration of absenteeism in the categories with a higher level of schooling.

The studies that address this issue show that sickness absenteeism has always existed in institutions, and it requires daily efforts from managers and constant training, as well as the implementation of well-conducted programs to identify the causes of sickness absenteeism, in order to understand the phenomenon and facilitate strategies to deal with the problem.^11^

Based on the assumption that the health and quality of life of workers interfere with the quality of the service provided, managers must invest in fundamental issues for the adequacy of the work environment, such as equipment, technology, and, especially, investments in the health of their workers.^19^ Sickness absenteeism can be characterized, then, as a management problem that must be addressed by the human resources management, and managers must deal with this problem, facilitating the development of strategies that generate well-being for the employees.^11^

A limitation of this study concerns data collection, so it was not possible to obtain more information that would allow a broader characterization of sickness absenteeism in the company.

However, the results of the present study allowed a first approximation of the occupational health process in this healthcare service provider, being a starting point for other studies. These findings, along with others in the literature, show the importance of improving workers’ quality of life and, consequently, reducing sickness absenteeism, by allowing and expanding discussion spaces and valuing the workers’ autonomy in the organization of work, having the workers themselves and the thought on their practices as references.^30^ The problems identified in the study and the possible interventions based on them can be much more effective if everyone, managers and workers, are summoned to this debate.

## CONCLUSIONS

The highest percentages of sickness absenteeism were found in women and in those with diseases of the musculoskeletal system and connective tissue, as well as in the emergency room sections and in the roles of service agents and analysts. Considering the longest periods of absence, the most identified categories were older people, circulatory system diseases, work in the administration section, and the motorcycle courier position. A significant percentage of sickness absenteeism was identified in the company, and it is necessary that managers invest in strategies to adapt the work environment.
